# Genome-wide association for heat tolerance at seedling stage in historical spring wheat cultivars

**DOI:** 10.3389/fpls.2022.972481

**Published:** 2022-08-25

**Authors:** Muhammad Ibrar Khan, Zarnishal Kainat, Saman Maqbool, Ambreen Mehwish, Suhaib Ahmad, Hafiz Muhammad Suleman, Zahid Mahmood, Mohsin Ali, Abdul Aziz, Awais Rasheed, Huihui Li

**Affiliations:** ^1^Department of Plant Sciences, Quaid-i-Azam University, Islamabad, Pakistan; ^2^Institute of Molecular Biology and Biotechnology, Bahauddin Zakariya University, Multan, Pakistan; ^3^Crop Sciences Institute, National Agricultural Research Centre (NARC), Islamabad, Pakistan; ^4^Institute of Crop Sciences, Chinese Academy of Agricultural Sciences (CAAS) & CIMMYT-China Office, Beijing, China; ^5^Nanfan Research Institute, CAAS, Sanya, Hainan, China; ^6^International Maize and Wheat Improvement Center (CIMMYT), Pakistan Office, NARC, Islamabad, Pakistan

**Keywords:** heat stress, heat stress tolerance, quantitative trait loci, quantitative trait nucleotides, single nucleotide polymorphism markers

## Abstract

Increasing global temperature has adverse effects on crop health and productivity at both seedling and reproductivity stages. It is paramount to develop heat tolerant wheat cultivars able to sustain under high and fluctuating temperature conditions. An experiment was conducted to characterize 194 historical wheat cultivars of Pakistan under high temperature at seedling stage to identify loci associated with heat tolerance using genome-wide association studies (GWAS). A quantitative trait locus, *TaHST1*, on chr4A was also characterized to identify the haplotypes at this locus associated with heat tolerance in wheat from Pakistan. Initially, the diversity panel was planted under control conditions (25°C/20°C day and night temperature) in a glass house. At three leaf stage, plants were subjected to heat stress (HS) by increasing temperature (40°C/35°C day and night), while one treatment was kept at control condition. After 7 days of HS, data were collected for seedling morphology. Heat stress reduced these traits by 25% (root weight) to 40% (shoot weight), and shoot biomass was largely affected by heat stress. A GWAS model, fixed and random model circulating probability unification (FarmCPU), identified 43 quantitative trait nucleotides (QTNs) on all chromosomes, except chr7B, were associated under both HS and control conditions. Thirteen QTNs were identified in control, while 30 QTNs were identified in HS condition. In total, 24 haplotypes were identified at *TaHST1* locus, and most of the heat tolerant genotypes were assigned to Hap-20 and Hap-21. Eleven QTNs were identified within 0.3–3.1 Mb proximity of heat shock protein (HSP). Conclusively, this study provided a detailed genetic framework of heat tolerance in wheat at the seedling stage and identify potential genetic regions associated with heat tolerance which can be used for marker assisted selection (MAS) in breeding for heat stress tolerance.

## Introduction

Bread wheat (*Triticum aestivum* L.) is the most widely farmed cereal grain crop, accounting for one-fifth of the calories consumed worldwide (Shahinnia et al., 2016). It is primarily farmed as a food source for humanity, feeding over 35% of the world’s population (Tahmasebi et al., 2013). According to the UN Food and Agriculture Organization (FAO), global wheat production is projected to exceed 761.7 million tonnes in 2020 (FAO, 2020). It is estimated that annual cereal production must increase by nearly 1 billion tonnes to feed the projected population of 9.1 billion by 2050.

Wheat is widely grown in the tropical and subtropical regions of the world, which are subjected to a variety of biotic and abiotic stresses. High temperature is one of the abiotic stresses which drastically affects the production of wheat (Rahaie et al., 2013). Global climate models reported that the average ambient temperature is expected to rise by 6°C by the end of the twenty-first century (De Costa, 2011). Many trials have revealed considerable yield losses in wheat due to HS, and it is anticipated that global wheat yields will drop by 4.1%–6.4% for every 1°C increase in global temperature (Liu et al., 2016). High temperature damages the wheat by effecting its physiological, biological and biochemical processes (Asseng et al., 2015). Heat stress affects the plant by damaging its photosynthetic machinery, compromised seed germination, reduce grain filling time duration, decrease in grain number, inactivation of Rubisco enzyme, slower the transportation of nutrients, premature leaf senescence, and reduce chlorophyll content which results in the reduction of yield (Hossain et al., 2013). Starch and protein content of grains are also affected by HS. Heat stress causes the generation of reactive oxygen species (ROS), which leads to membrane instability, lipid peroxidation, protein oxidation, and nucleic acid damage (Mishra et al., 2011; Mittler et al., 2011).

Several genome-wide association studies (GWAS) and quantitative trait loci (QTL) mapping concluded that heat stress tolerance (HST) in wheat is polygenic and is influenced by environmental factors (Guan et al., 2018; El Hassouni et al., 2019; Li et al., 2019). Several major and minor QTL were identified in these studies at vegetative and reproductive stages. For example, five QTL were identified on chr1B, chr1D, chr2B, chr6A and chr7A for HST in a recombinant inbred line (RIL) population (Ventnor × Karl 92) by using AFLP, SSR and EST markers (Talukder et al., 2014). In another study, several QTL were detected at chr1A, chr1B, chr2B, chr3B, chr5A and chr6D for HST in a RIL population (Halberd × Cutter) at grain filling stage using HSI as phenotypic data for QTL mapping (Mason et al., 2010). A GWAS study in US winter wheat identified four loci at chr3B, chr7D and chr2A for shoot length and several loci were detected at chr2B, chr2D, chr4A, chr4B and chr5B for chlorophyll content at seedling stage under heat stress (Maulana et al., 2018). Recently, Lu et al. (2022) evaluated 48 wheat genotypes and concluded that genotypes tolerant at seedling stage showed higher yield at reproductive stage after heat treatment, and seedling evaluation can be used for early selection of heat tolerant wheat genotypes.

The genome size of hexaploid bread wheat is ~17Gb with 85% of repetitive sequence (IWGSC, 2018), frequent translocations, large deletions and inversions are found among wheat cultivars (Cheng et al., 2019; He et al., 2019). It is very challenging to isolate the genes responsible for HST in wheat by map-based cloning although heat tolerant mutants were identified 20 years ago (Mullarkey and Jones, 2000). *TaHST1* is a QTL that is significantly associated with HST in both vegetative and reproductive stages of wheat (Zhai et al., 2021). It was mapped on the distal terminal of chr4A. According to reference genome sequence of Chinese spring (CS), this 0.949 Mb region has 19 high confidence genes (IWGSC, 2018). Five markers *Xhau1-Xhau5* were used for the detection different haplotypes in this region in wheat cultivars (Zhai et al., 2021). Further investigation revealed that an unusual high number of deletion mutation in this region was observed, which was confirmed by using sequencing data from 10+ wheat genome project.^1^

This study was designed to (i) characterize a diversity panel consisting of historical spring wheat cultivars against heat stress at seedling stage, (ii) identify quantitative trait nucleotides (QTNs) associated with heat tolerance at seedling stage in historical spring wheat cultivars of Pakistan, and (iii) characterize *TaHST1* locus in diversity panel using gene-specific markers for its association with HST at seedling stage.

## Materials and methods

### Germplasm

The germplasm used in this study consists of a panel of 194 historical bread wheat cultivars of Pakistan released in the years between 1911 and 2019. Each cultivar with its release year and pedigree is given in Supplementary Table 1.

### Phenotyping at seedling stage

Screening of seedlings against HS was performed at National Agriculture Research Centre, Islamabad, Pakistan. For this experiment, small transparent glasses (3 inch × 6 inch) filled with a mixture of peat moss and soil (80%:20%) were used. Six seeds of each cultivar were sown in a single glass. Three glasses of each cultivar in randomization were used for experimentation in each treatment, i.e., control and heat stress (HS). Before sowing, seeds were surface sterilized with 2% NaOCl. During the whole experiment, the plants were watered regularly to ensure that there will be no drought stress. At three leaves stage after germination, one panel was kept in control conditions with 25°C/20°C day/night temperature, respectively. The 20 days old seedlings were subjected to HS by applying 40°C/35°C day and night time temperature, respectively. After 7 days of stress treatment, root length (RL), shoot length (SL), root weight (RW), and shoot weight (SW) was measured. A total of six measurements were taken from each line and then average data was used for further statistical analysis. Biomass of both control and stressed plants was taken by electrical weighing balance.

Heat susceptible index of all traits in optimal and stress condition was calculated by formula proposed by Fisher and Maurer (1978):


HSI=[1−YD/YP]/D

where YD is the mean of genotypes in heat stress condition, YP is the mean of genotypes in optimal conditions, D = 1 − [mean of genotypes in stress condition/mean of genotypes in control condition].

### DNA extraction and genotyping

DNA from each cultivar was extracted following standard protocol (Dreisigacker et al., 2013). Wheat cultivars were genotyped using 50 K SNP array, which generated 66,876 SNPs. After filtering with minor allele frequency of >5% and missing data of <10%, a total of 52,610 SNPs were retained and used for the GWAS analysis.

### Genotyping for *TaHST1* locus

The details of primers used in the reaction are described in Supplementary Table 2. The PCR reaction mix (10 μl) consisted of 3 μl PCR H_2_O, 5 μl master mix (2× Taq PCR mix), forward and reverse primers 0.5 μl each and 1 μl of DNA. The PCR was carried out at following conditions: initial denaturation at 95°C for 5 min (1 cycle), 94°C for 1 min, annealing at 65°C, 56°C, 58°C, 65°C, and 60°C, respectively, for 1 min, extension at 72°C for 1 min (35 cycles). The PCR products were checked in 2% agarose gel.

### Statistical analysis

Pearson’s correlation coefficient (*r*) among all traits was used to examine the correlation between different traits in control and HS conditions. This statistical analysis was performed by using R statistical software. Genotypic data from the 50 K SNP array was subjected to quality control for further use. Initially, all the SNPs with missing data >10% and minor allele frequency (MAF) <5% were excluded. The remaining SNPs were used for GWAS.

Principal component analysis (PCA) was performed to get information about principal structure and first five PC scores were taken as a Q matrix. Kinship matrix (k) was calculated by TASSEL v5.1. FarmCPU model was used to identify the quantitative trait nucleotides (QTNs). The GWAS was carried out using R-Package of Rmvp v3.1. Linkage disequilibrium (LD) was determined between SNPs identified as QTNs. To check the linkage disequilibrium within and across three genomes of bread wheat (A, B and D), squared allele frequency correlation (*r*^2^) values were used between marker pairs. Markers containing (*r*^2^) value 1 on the same chromosome were removed.

## Results

### Phenotypic data analysis

**Figure 1:**
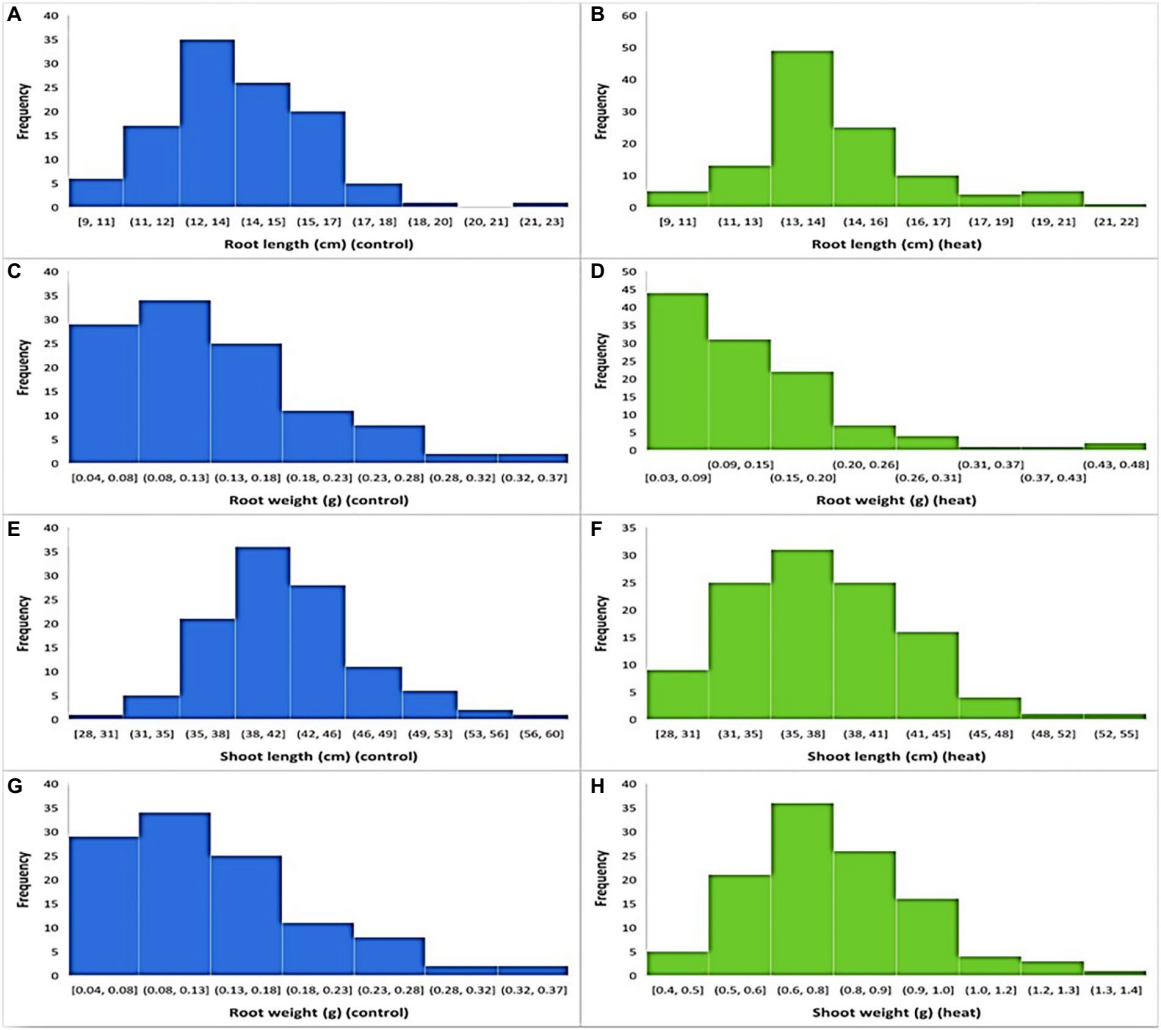
Frequency distribution of traits at optimal and heat stress condition. **(A,B)** shows root length at optimal and HS conditions, while **(C,D)** shows root weight, **(E,F)** shoot length, and **(G,H)** shows shoot weight at optimal and heat stress conditions, respectively.

In both temperature treatments (control and HS), significant variations in seedling phenotypes were observed in the diversity panel. The descriptive statistics and frequency distribution plots under control and HS conditions are given in Table 1; Figure 1, respectively. Mean RL at control condition was 13.74 cm with a range from 9.3 cm (Raj) to 22.3 cm (C-250), while under HS, the mean RL was 14.14 cm with a range from 7 cm (AZRC) to 21.5 cm (Lasani-08). In control condition, the mean RW was 0.125 g with a range from 0.03 (Raj) g to 0.5 g (Sutluj-86), while in HS condition the mean RW was 0.12 g with a range from 0.01 (AZRC) to 0.45 g (Takbeer). Mean SL was 40.75 cm with a range of 26 cm (Dilkash) to 59 cm (Faisalabad-83) compared with HS condition where mean SL was 37 cm with a range from 24 cm (Nishan-21) to 49 cm (NIA-Sunder). Similarly, mean SW at control condition was 1.5 g with a range from 0.5 g (Pakhtunkhawa-15) to 3 g (Rashkoh-05) and in HS condition mean SW was 0.7 g with a range of 0.3 g (Subhani) to 1.4 g (Takbeer). In HS, SL was reduced by 15%–20%, and SW was reduced by 40%–45%. RL and RW were only reduced by 10%–15% each. Fold variation was observed in HS treatment. Mean fold variation for RL was 1.02-fold ranging from 0 to 2-fold. In RW, there was 3-fold variation ranging from 1 to 5.4-fold. Similarly, mean fold variation for SL in HS was 0.42-fold ranging from 0.006 to 1.0-fold.

**Table 1:** Descriptive statistics of seedling traits of historical wheat cultivars at seedling stage under control and heat stress condition.

Traits	Control			HS^a^			HSI^b^		Fold increase (HS)^c^	
	Mean	Range	SD	Mean	Range	SD	Mean	Range	Mean	Range
Root length (cm)	13.74	9.3–23.3	2.1	14.14	7–21.5	2.12	1.64	−1.5 to 4.7	1.02-fold	0.1-2-fold
Root weight (g)	0.125	0.03–0.5	0.07	0.12	0.01–0.45	0.07	−0.24	−7.5 to 1.3	3-fold	0.1–5.4-fold
Shoot length (cm)	40.75	26–59	5.2	37	24–49	4.5	0.99	−10 to 5.3	0.42-fold	0.006–1.0-fold
Shoot weight (g)	1.5	0.5–3	0.4	0.7	0.3–1.4	0.19	1.01	−1.3 to 2.4	1.43-fold	0.02–3.15-fold

a HS, heat stress.

b HSI, Heat susceptible index.

c Fold-increase, fold-increase in data value in heat stress treatment.

**Figure 2:**
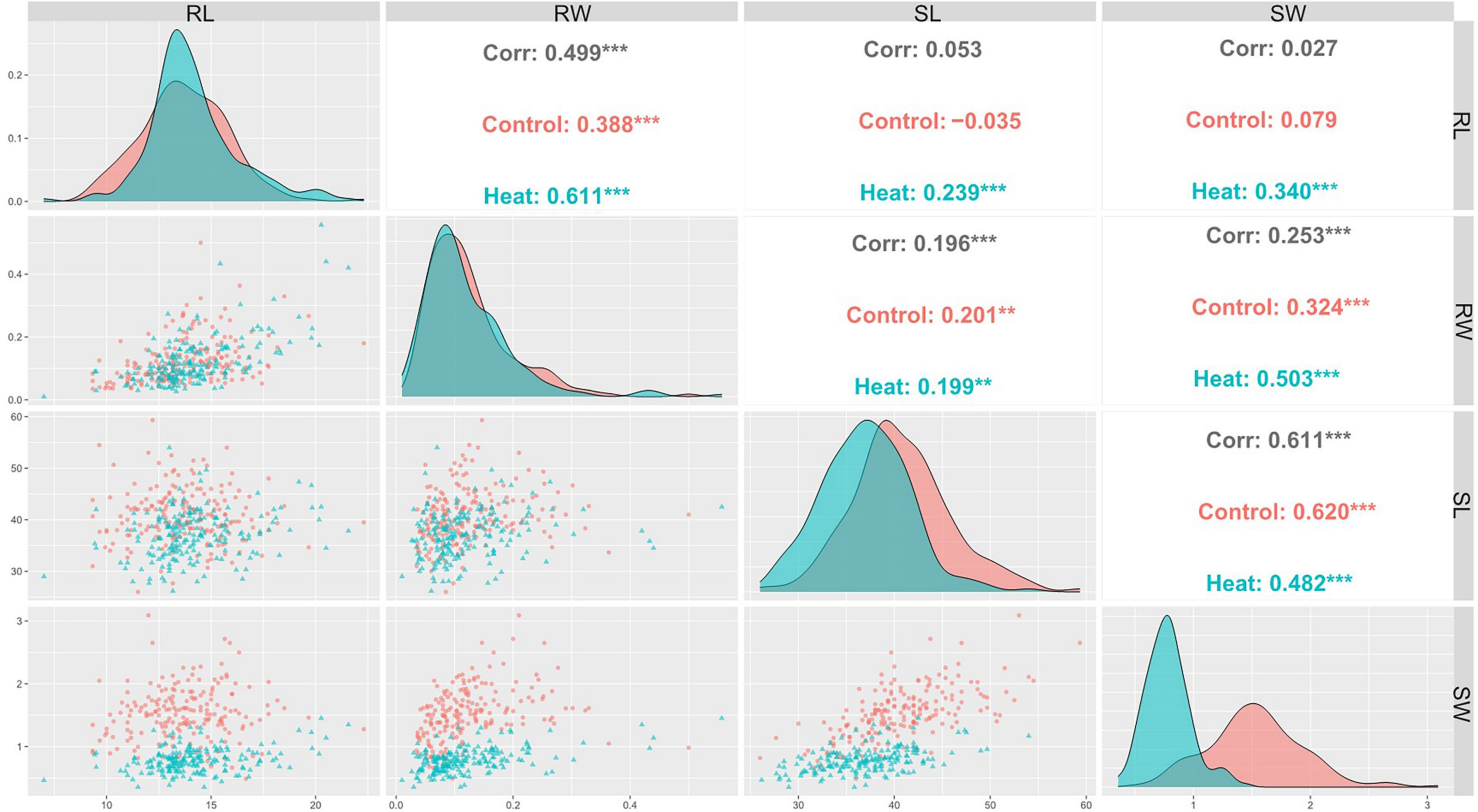
Pearson’s correlation coefficients describing association of various traits in wheat under control and HS conditions.

Pearson coefficient of correlation between all traits under control and HS conditions are shown in Figure 2. Positive correlation was observed between RL and RW under control (*r* = 0.39) and HS (*r* = 0.61) conditions. Correlation between RW and SW was (*r* = 0.32) and (*r* = 0.5) under control and HS, respectively. While RW and SL had (*r* = 0.2) and (*r* = 0.19) in control and HS, respectively. SL and SW also showed significantly positive correlation of in control (*r* = 0.62) and HS (*r* = 0.42).

Mean values of all traits were used to calculate heat susceptible index (HSI). Almost 7.7% genotypes showed HSI < 0.5 for SW and 23% genotypes showed HSI < 0.5 for SL. Phenotypic data of all 194 wheat cultivars used in this study under control and heat stress conditions is given in Supplementary Table 4.

### SNP marker distribution and statistics

In total 66,836 SNP markers were genotyped with 50 K SNP array. After quality control, 38 markers were removed having missing data and 14,188 markers were removed with MAF <0.05. Subsequently, 52,610 SNPs were used for GWAS. The SNPs were distributed on all chromosomes with maximum number of SNPs on chr2B (*n* = 3,510) and least number of SNPs on chr4D (*n* = 1,210). Highest number of SNPs were distributed on B-genome (20,636) followed by (18,564) on A-genome and (13,408) on D-genome. Genotypic data as HapMap file for all wheat cultivars used in this study is given in Supplementary Table 3.

### QTNs associated with HS tolerance at seedling stage

**Figure 3:**
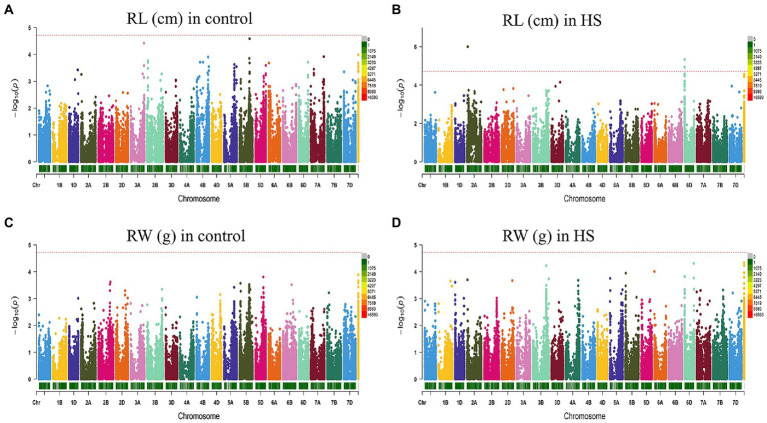
Manhattan plot showing density of SNP markers associated with root length **(A)** control condition; **(B)** HS condition, root weight **(C)** control condition; **(D)** HS condition.

**Figure 4:**
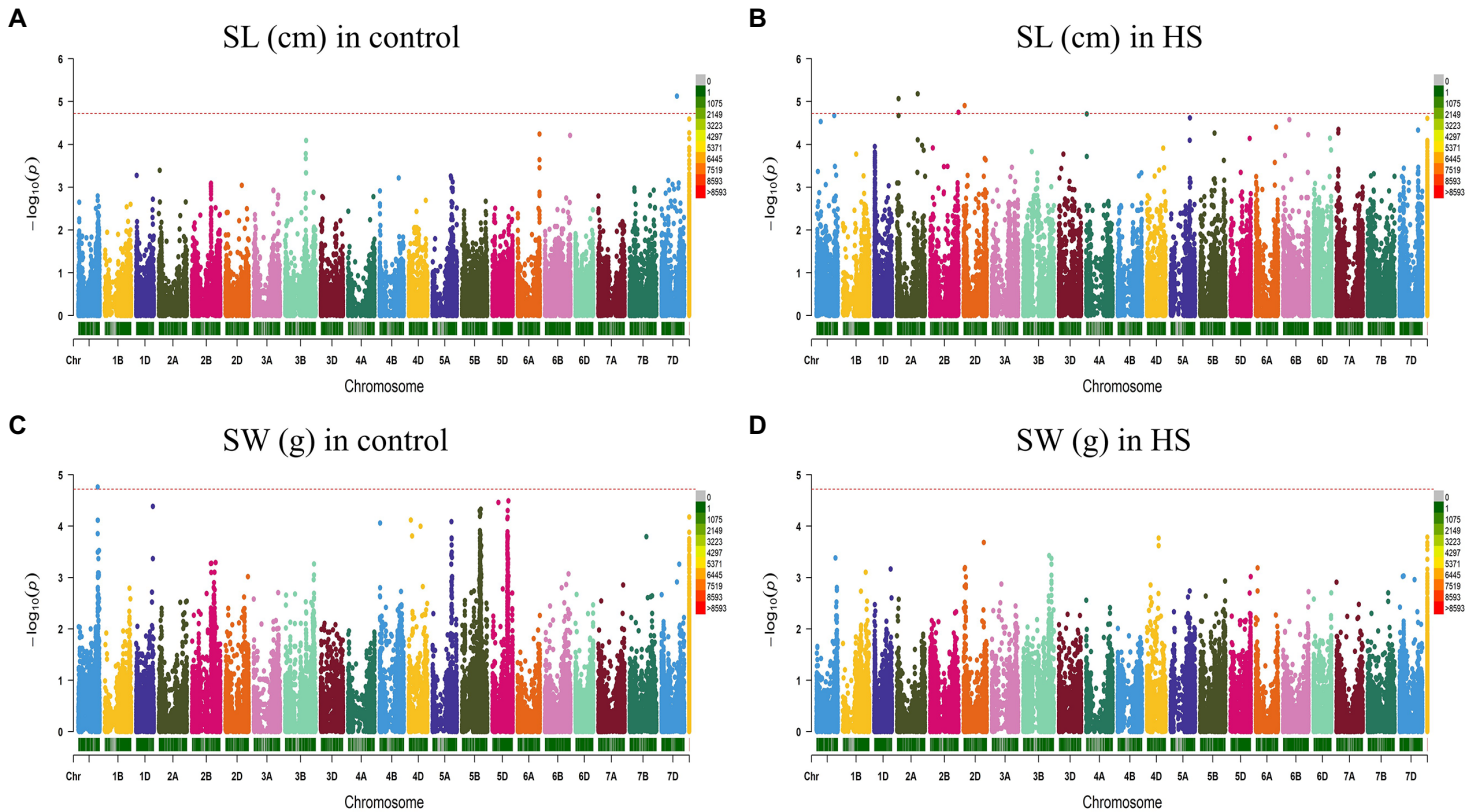
Manhattan plot showing density of SNP markers associated with shoot length **(A)** control condition; **(B)** HS condition, shoot weight **(C)** control condition; **(D)** HS condition.

FarmCPU model has the high statistical power to control the false positive therefore we used this model in our study to report QTNs associated with phenotypes. Manhattan plots of all traits under study are presented in Figures 3, 4. SNP markers which were significantly associated with the traits in control and HS conditions are given in Table 2. In total 43 QTNs were identified that were associated with all four traits in control (*n* = 13) and HS conditions (*n* = 30). For RL in control condition, two QTNs were identified on chr3A at 721.3 Mb and chr5B at 559.4 Mb (Figure 3A; Table 2). Three QTNs were identified on chr2A, chr3D and chr6D for RL in HS condition (Figure 3B; Table 3).

**Table 2:** Quantitative Trait Nucleotides (QTNs) associated with RL, RW, SL and SW at seedling stage in control and HS conditions.

Condition	Traits	SNP	Chr	Position	MAF	Value of *p*	Effect
Control	RL	AX-112287935	3A	721.39	0.3	3.79E-05	−1
		AX-95684632	5B	549.4	0.2	2.59E-05	1.4
	SL	AX-109325061	3B	594.47	0.4	8.17E-05	2.1
		AX-108727314	6A	615.42	0.1	5.73E-05	3.7
		AX-86169320	6B	711.29	0.1	5.93E-05	4.3
		AX-110237200	7D	434.43	0.1	7.52E-06	4.6
	SW	AX-95653494	1A	548.04	0.3	1.73E-05	0.2
		AX-179558207	1D	473.96	0.3	4.13E-05	0.2
		AX-111708768	4B	15.5	0.1	8.68E-05	0.3
		AX-94596238	5B	532.42	0.4	4.67E-05	0.2
		AX-86179100	5B	495.8	0.3	5.02E-05	0.2
		AX-94582897	5D	457.68	0.5	3.22E-05	0.2
		AX-110425904	5D	171.09	0.5	3.46E-05	−0.2
HS	RL	AX-111504604	2A	46.4	0.1	9.80E-07	2.4
		AX-179558694	3D	462.53	0.2	7.17E-05	2
		AX-108843268	6D	0.87	0.1	4.63E-06	1.7
	RW	AX-111026550	3B	720.85	0.2	5.90E-05	0
		AX-110621537	6A	1.87	0.1	9.79E-05	0.1
		AX-86183895	6D	491.99	0.1	4.89E-05	0.1
	SL	AX-86167844	1A	500.83	0.1	2.13E-05	4.2
		AX-94782852	1A	120.7	0.1	2.95E-05	4.8
		AX-108843795	2A	585.71	0.1	6.58E-06	4.7
		AX-94770627	2B	803.19	0.1	1.78E-05	−4.9
		AX-94530046	2D	29.26	0.5	1.25E-05	2.7
		AX-94594742	4A	17.45	0.1	1.94E-05	4.5
		AX-94750554	5A	543.34	0.1	2.39E-05	4.4
		AX-94633634	5B	408.03	0.3	5.45E-05	2.8
		AX-94490118	5D	546.55	0.1	7.26E-05	3.2
		AX-111242222	6A	571.46	0.1	3.96E-05	4.3
		AX-108856108	6B	181.67	0.1	2.65E-05	4.5
		AX-95156239	6D	463.26	0.1	7.19E-05	4.6
		AX-108774450	7A	55.9	0.1	4.47E-05	3.5
		AX-109063499	7D	519.19	0.1	4.64E-05	4.7
	SW	AX-94794804	1A	536.37	0.1	4.16E-04	0.1
		AX-95654436	1B	647.18	0.1	7.93E-04	0.1
		AX-95104531	1D	461.77	0.3	6.83E-04	−0.1
		AX-179475749	2D	29.15	0.3	6.76E-04	0.1
		AX-179558144	2D	569.41	0.1	2.07E-04	0.1
		AX-109576124	3B	733.29	0.1	3.74E-04	0.1
		AX-111862796	4D	325.81	0.5	2.39E-04	−0.1
		AX-94643729	5D	576.15	0.4	9.59E-04	−0.1
		AX-179558555	6A	48.85	0.1	6.48E-04	0.1
		AX-95155896	7D	88.55	0.2	9.57E-04	0.1

**Table 3:** HSP genes associated with QTNs identified in HS.

Trait	SNP	CHR	Distance from HSP (Mb)	Annotation	Gene ID
SL	AX-86167844	1A	3	TaHSP100.1	TraesCS1A02G304800
SW	AX-94794804	1A	−1.44	TaHSP40.10	TraesCS1A01G349900
SW	AX-95654436	1B	−0.99	TaHSP40.26	TraesCS1B01G423600
RW	AX-111026550	3B	3.17	TaHSP40.89	TraesCS3B02G470100
RW	AX-111026550	3B	−3.14	TaHSP40.90	TraesCS3B02G475500
RL	AX-179558694	3D	−0.31	TaHSP70.37	TraesCS3D02G351900
RL	AX-179558694	3D	−0.65	TaHSP70.38	TraesCS3D02G352400
SW	AX-111862796	4D	2.99	TaHSP40.133	TraesCS4D01G189000
SL	AX-111242222	6A	−3.03	TaHSP40.194	TraesCS6A02G341900
RW	AX-110621537	6A	−0.52	TaHSP60.54	TraesCS6A02G006400
SL	AX-95156239	6D	0.8	TaHSP60.69	TraesCS6D02G383500

“−” and “+” sign in distance from HSP indicates presence of SNP in upstream and downstream, respectively.

For RW in control conditions, no significant QTN was identified. In HS conditions, three QTNs were identified for RW on chr3B (720.85 Mb), chr6A and chr6B (Figure 3D; Table 2). In control condition, four QTNs were identified for SL on chr3B, chr6A, chr6B and chr7D. All these QTNs were mapped at 594.59 Mb, 615.41 Mb, 711.28 Mb and 443.42 Mb, respectively and each QTN was represented by one SNP (Figure 4A; Table 2). Fourteen QTNs were identified for SL in HS condition (Figure 4B; Table 2). For SW in control condition, 7 QTNs were identified at chr1A, chr1D, chr4B, chr5B and chr5D (Figure 4C; Table 2). In HS condition, 10 QTNs were identified for SW (Figure 4D; Table 2).

**Figure 5:**
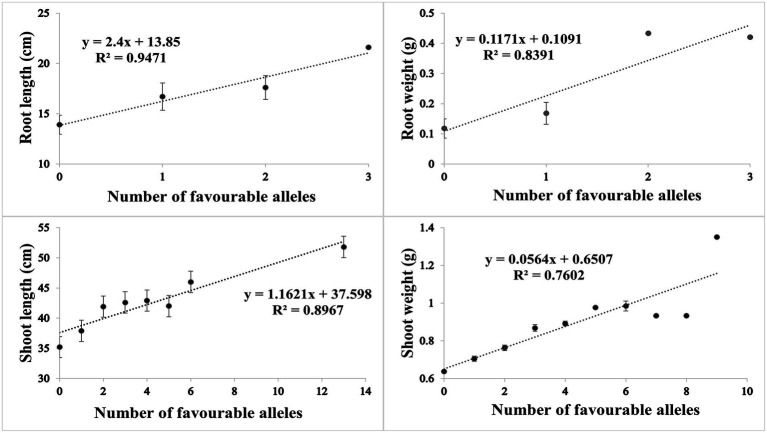
Scatter plots showing the effect of favorable alleles in **(A)** root length, **(B)** root weight, **(C)** shoot length and **(D)** shoot weight in HS.

**Figure 6:**
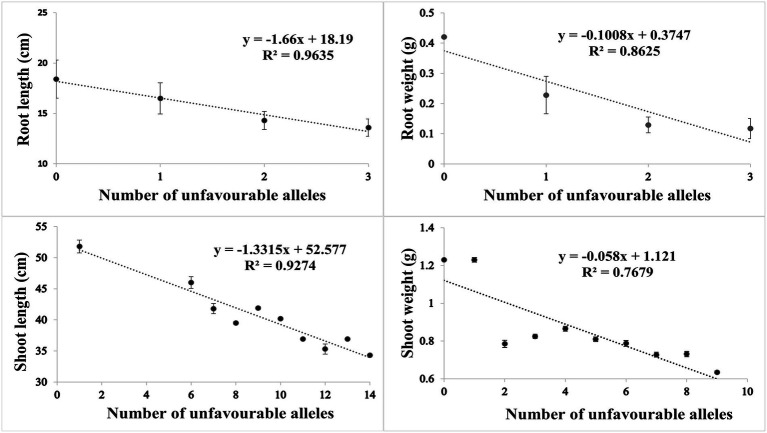
Scatter plots showing the effect of unfavorable alleles in **(A)** root length, **(B)** root weight, **(C)** shoot length and **(D)** shoot weight in HS.

Based on SNP effect, favorable and unfavorable alleles were identified, and their frequencies were determined. For RL under control conditions, 43 (38%) cultivars did not have any of the favorable allele, while five cultivars had maximum number of two favorable alleles. Similarly, four cultivars (3.6%) did not have unfavorable allele while 59 cultivars (53.1%) had maximum number of three unfavorable alleles. For RW under control condition, 81 (71%) cultivars had no favorable allele, while 30 (26%) cultivars had one favorable allele. Similarly in heat stress condition, only one cultivar had no unfavorable allele, while maximum number of 69 (61%) cultivars had three unfavorable alleles. For SL in control condition, 60 (53%) cultivars had no favorable allele while six cultivars had more than three favorable alleles. Similarly, in HS two cultivars had one unfavorable allele and maximum number of 17 cultivars had 14 unfavorable alleles. For SW in control, 40 (35%) had no favorable allele, while 5 (4%) cultivars had maximum seven favorable alleles. Similarly, in HS condition, 20 (17%) cultivars had no unfavorable allele while maximum number of 92 (81%) cultivars had one unfavorable allele. The coefficient of determination (*R*^2^) indicated that effect of favorable alleles ranged from *R*^2^ = 0.94 (RL) to *R*^2^ = 0.76 (SW; Figures 5A–D), while effect of unfavorable alleles ranged from *R*^2^ = 0.96 (RL) to *R*^2^ = 0.76 (SW; Figures 6A–D).

In wheat, the position of all heat shock proteins (HSPs) were identified according to Kumar et al. (2020). In total, 11 QTNs were identified in proximity of HSPs (Table 3). The closet QTN was for RL (AX-179558694) on chr3D which was 0.31 Mb close to HSP in upstream region. The same QTN was 0.65 Mb away from another HSP in upstream region. Another QTN for RW (AX-111026550) on chr3B was present between two HSPs at 3.14 Mb and 3.17 Mb away in upstream and downstream region, respectively. Three QTNs for SL (AX-111242222, AX-86167844, and AX-95156239) on chr6A, chr1A and chr6D were present at 3.03 Mb, 3.0 Mb and 0.80 Mb in upstream and downstream region, respectively. On chr6A, QTN for RW (AX-110621537) was present at 0.52 Mb in upstream region. For SW three QTNs (AX-94794804, AX-95654436, and AX-111862796) on chr1A, chr1B and chr 4D at 1.43 Mb, 0.99 Mb and 2.99 Mb in upstream and downstream region, respectively.

### Characterization of *TaHST1* locus in diversity panel

A locus on terminal end of chr4A consisting of 0.949 Mbp plays an important role in heat stress tolerance in wheat and likely to have a heat stress tolerance gene (Zhai et al., 2021). This region contains 19 high confidence genes and was characterized by using five gene-specific DNA markers (Zhai et al., 2021). Among them, *Xhau-1*, -*2, -3,* and *5* were dominant markers, while *Xhau-4* was either co-dominant or dominant depending on the lines analyzed. In total, 24 haplotypes in historical bread wheat cultivars of Pakistan were identified based on the allelic variation of five markers (Table 4). Haplotype 1 has highest frequency of 19.4% with 3 deleted sites followed by Hap2 (13.1%) with 5 deleted sites and Hap17 (11.4%) with 2 deleted sites. Twenty-three (13%) cultivars showed complete deletion of region as no amplification was observed with any marker, whereas 17 cultivars (9.7%) were amplified with all markers used in the study.

**Table 4:** Description of 24 haplotypes for *TaHST1* QTL.

Haplotype	Xhau-1	Xhau-2	Xhau-3	Xhau-4	Xhau-5	Deleted sites	Number of lines	Frequency (%)	RL (cm) in HS	RW (g) in HS	SL (cm) in HS	SW (g) in HS
Hap1	−	−	+	127	−	3	34	19.4	14.3	0.14	36.4	0.8
Hap2	−	−	−	−	−	5	23	13.1	14.3	0.11	37.4	0.78
Hap3	−	−	+	127	+	2	20	11.4	13.7	0.1	37.6	0.84
Hap4	−	−	−	195	−	4	17	9.7	14.1	0.12	38.8	0.75
Hap5	+	+	+	127	+	0	16	9.1	15	0.15	37.3	0.83
Hap6	−	−	−	127	+	3	11	6.3	15.3	0.17	38	0.83
Hap7	−	−	−	127	−	4	9	5.1	9.4	0.09	35.3	0.87
Hap8	−	−	−	127	−	4	9	5.1	13.6	0.09	37	0.72
Hap9	+	−	+	127	+	1	8	4.6	12.8	0.09	36.4	0.73
Hap10	+	+	+	127	−	1	7	4	15	0.13	41.1	0.85
Hap11	−	+	+	127	−	2	5	2.9	13.8	0.13	35	0.67
Hap12	−	−	−	195	+	3	5	2.9	15	0.11	36	0.82
Hap13	−	−	+	−	−	4	4	2.3	13.2	0.07	37.2	0.73
Hap14	−	−	−	−	+	4	3	1.7	12.7	0.07	31.2	0.66
Hap15	+	+	+	−	+	1	3	1.7	15.6	0.17	39.7	0.82
Hap16	+	+	−	127	−	2	2	1.1	14.7	0.13	35.6	0.71
Hap17	−	+	−	−	−	4	1	0.6	20.2	0.17	40	0.88
Hap18	+	+	+	−	−	2	1	0.6	14.3	0.26	42	0.59
Hap19	+	−	+	−	+	2	1	0.6	13.2	0.14	43	0.76
Hap20	+	+	+	195	+	0	1	0.6	14	0.11	40.6	1.03
Hap21	−	+	−	127	−	3	1	0.6	14	0.22	41.3	1
Hap22	−	+	−	127	+	2	1	0.6	13.4	0.07	32	0.7
Hap23	−	−	+	195	−	3	1	0.6	12.5	0.09	36.3	0.7
Hap24	−	−	+	195	+	2	1	0.6	14.3	0.07	36	0.8
Total							175	100				

+ and − shows positive and negative amplifications. Xhau-4 is a co-dominant marker showed amplifications of 127 bp or 195 bp depending on the genotype.

**Figure 7:**
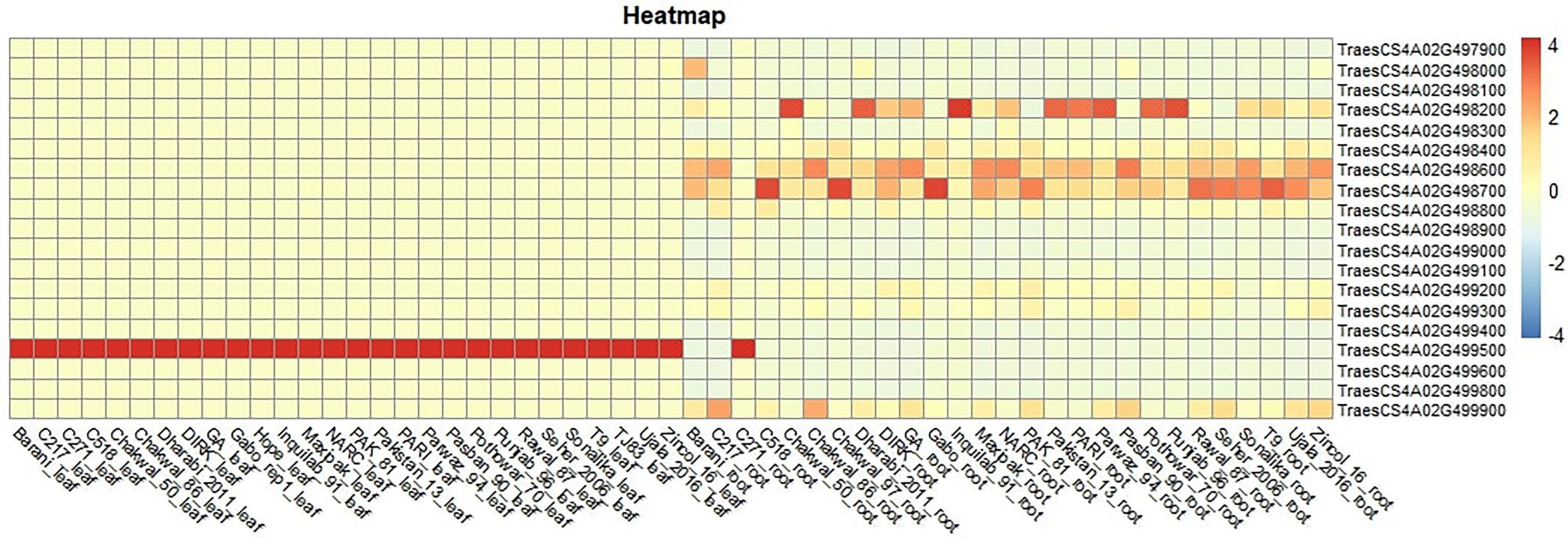
Expression of 19 genes in roots and leaves of a subset of 28 wheat cultivars used in this study.

The basal expression of these genes in different lines was observed using RNAseq data of 24 cultivars present in the diversity panel (unpublished data). Five genes were differentially expressed in roots, while only one gene (*TraesCS4A02G499500*) was differentially expressed in leaves (Figure 7). This gene is a cellular component of plant and resides in thylakoid membrane of chloroplast. At molecular level, this gene is involved in copper ion binding and electron transfer activity. Higher expression of the gene was observed in Punjab-96, Pothowar-7, Parwaz-94, Pari, Inquilab-91, Dharabi-2011 and chakwal-50.

## Discussion

In present study, a diversity panel consisting of 194 historical wheat cultivars was evaluated for heat stress tolerance and identification of loci associated with RL, RW, SL, and SW under HS tolerance. The panel used in this study for association mapping showed great variations in RL, RW, SL, and SW at control and HS conditions.

### HSI of historical wheat cultivars

Significant variations in all traits were observed in both control and HS conditions. The cultivars having HSI higher than 0.5 were regarded as sensitive to HS, while those having lower HSI values were heat tolerant (Fisher and Maurer, 1978). More than 75% cultivars showed 0–1 HSI values, and modern cultivars like Markaz-19 and MA-21 were highly sensitive to HS. Sutluj-86, Bahawalpur-97 and Pirsabak-15 showed very low HSI values. Genotypes with minimum HSI values for SL were Bahawalpur-97, Pirsabak-15 and Sutluj-86. For SW minimum HSI values were shown by Sutluj-86, Bahawalpur-97 and Pirsabak-15. Based on these results, these three cultivars were considered as heat tolerant as compared to other genotypes.

The analysis indicated that all the traits are highly co-related with each other. No significant correlation was found previously between these traits (Ram Poudel et al., 2021). Under HS, RL and RW were significantly correlated to SL and SW. The higher positive correlation indicated that HS effect on the below-ground parts can be selected based on the above-ground shoot traits.

To the best of our knowledge, very few GWAS have been conducted for HST at seedling stage in wheat. Maulana et al. (2018) evaluated US winter wheat cultivars and assessed HS on traits like leaf chlorophyll content, SL, number of leaves per seedling and seedling recovery. However, many gene mapping studies were conducted for heat stress tolerance at flowering and reproductive stages (Vijayalakshmi et al., 2010; Talukder et al., 2014). In control condition, the QTNs associated with RL on chr3A and chr5B were not found in stress condition. At seedling stage, very little is known for MTAs associated with roots under heat stress in wheat. In HS, two QTNs were identified on chr2D for SL and SW at 29 Mb which are located in close proximity of 4 QTL identified by Sangwan et al. (2019) on chr2D at 27.9 cM for days to heading, days to maturity and photosynthetic rate.

In our findings, common QTNs associated with SL were found in both control and heat stress conditions. The presence of QTNs on chr6A, chr6B and chr7D for SL in control and HS conditions indicated that these QTL are strictly related to SL and have no effect of HS. Contrary to Maulana et al. (2018), no QTN on chr3B and chr7B for SL in control and HS conditions.

Under control, QTN associated with SL were positioned on chr1A, chr1D, chr4B, chr5B and chr5D. These QTNs were not found under HS conditions. No QTN was identified on chr3B for SW in HS. In a previous study, a QTL on chr3B chromosome was found responsible for increase shoot biomass in heat stress condition not found in this study (Thomelin et al., 2021).

*TaHST1* locus plays a significant role in heat stress tolerance at both seedling and reproductive stage. Twenty-four haplotypes were detected using a set of five primers to detect *TaHST1* locus Among the most frequent haplotypes, hap-20 had the highest SW values followed by hap-21. The results indicated that the presence of *TaHST1* has significant effects on wheat in heat stress condition, rather than optimal conditions. The genes that are present in this region has higher expression in roots, and only one gene involved in copper ion binding and electron transfer activity was highly expressed in leaves. It is likely that this gene is an important component of this locus and is associated with SL in HS conditions.

## Conclusion

In a nutshell, some of the QTNs that are related to heat stress tolerance found in this research were already identified by other studies, although the stages of development were different in the previous and current study. Many new QTNs were also identified that are significantly related to HST. Wheat cultivars having favorable haplotypes of *TaHST1* locus could be promising candidates for breeding for heat stress adaptability. To the best of our knowledge, this is first GWAS on historical wheat cultivars of Pakistan at seedling stage. The significant SNP markers and cultivars identified in this study will be used for marker assisted selection (MAS) for heat tolerant to facilitate the trait selection during breeding.

## Data availability statement

The phenotypic and genotypic data presented in this article can be found in Supplementary material. The RNAseq data presented in this article can be accessed from NCBI BioProject ID PRJNA863398. Further inquiries can be directed to corresponding authors.

## Author contributions

MK, ZK, and SM conducted greenhouse experiment and prepared manuscript. AM, SA, and HS assisted in data collection. ZM, MA, and AA performed GWAS analyses. AR and HL designed the experiments and finalized the manuscript. All authors contributed to the article and approved the submitted version.

## Funding

This work was supported by the National Science Foundation of China (32022064) and the Project of Hainan Yazhou Bay Seed Laboratory (B21HJ0223).

## Conflict of interest

The authors declare that the research was conducted in the absence of any commercial or financial relationships that could be construed as a potential conflict of interest.

## Publisher’s note

All claims expressed in this article are solely those of the authors and do not necessarily represent those of their affiliated organizations, or those of the publisher, the editors and the reviewers. Any product that may be evaluated in this article, or claim that may be made by its manufacturer, is not guaranteed or endorsed by the publisher.

## Footnotes

^1^ http://www.10wheatgenomes.com/

## References

[ref1] Asseng S. Ewert F. Martre P. Rötter R. P. Lobell D. B. Cammarano D.. (2015). Rising temperatures reduce global wheat production. Nat. Clim. Chang. 5, 143–147. doi: 10.1038/nclimate2470

[ref5] Cheng H. Liu J. Wen J. Nie X. Xu L. Chen N.. (2019). Frequent intra-and inter-species introgression shapes the landscape of genetic variation in bread wheat. Genome Biol. 20:136. doi: 10.1186/S13059-019-1744-X, PMID: 31300020 PMC6624984

[ref6] De Costa W. A. J. M. (2011). Review of the possible impacts of climate change on forests in the humid tropics. J. Natl. Sci. Found. 39, 281–302. doi: 10.4038/JNSFSR.V39I4.3879/GALLEY/3136/DOWNLOAD

[ref01] Dreisigacker S. Tiwari R. Sheoran S. (2013). Laboratory manual: ICAR-CIMMYT molecular breeding course in wheat. Haryana: Directorate of Wheat Research Karnal, India.

[ref8] FAO (2020). Crop prospects and food situation – Google Scholar 2019. Annual Report to CGIAR Consortium Wageningen, the Netherlands. CGIAR Research Program on Climate Change, Agriculture and Food Security (CCAFS).

[ref9] Fisher R. A. Maurer R. (1978). Drought resistance in spring wheat cultivars. I. Grain yield responses. Aust. J. Agric. Res. 29, 897–912. doi: 10.1071/AR9780897

[ref10] Guan P. Lu L. Jia L. Kabir M. R. Zhang J. Lan T.. (2018). Global QTL analysis identifies genomic regions on chromosomes 4A and 4B harboring stable loci for yield-related traits across different environments in wheat (*Triticum aestivum* L.). Front. Plant Sci. 9:529. doi: 10.3389/fpls.2018.00529, PMID: 29922302 PMC5996883

[ref7] Hassouni K. El Belkadi B. Filali-Maltouf A. Tidiane-Sall A. Al-Abdallat A. Nachit M.. (2019). Loci controlling adaptation to heat stress occurring at the reproductive stage in durum wheat. Agronomy 9:414.

[ref11] He F. Pasam R. Shi F. Kant S. Keeble-Gagnere G. Kay P.. (2019). Exome sequencing highlights the role of wild-relative introgression in shaping the adaptive landscape of the wheat genome. Nat. Genet 51, 896–904. doi: 10.1038/s41588-019-0382-2 31043759

[ref12] Hossain A. Sarker M. A. Z. Saifuzzaman M. da Silva J. A. T. Lozovskaya M. V. Akhter M. M. (2013). Evaluation of Growth, Yield, Relative Performance and Heat Susceptibility of Eight Wheat (*Triticum aestivum* L.) Genotypes Grown Under Heat Stress. Academia. Edu Available at: https://www.academia.edu/download/31190568/IJPP11211364758200.pdf (Accessed July 21, 2022).

[ref13] International Wheat Genome Sequencing Consortium (IWGSC) (2018). Shifting the limits in wheat research and breeding using a fully annotated reference genome. Science 361:eaar7191. doi: 10.1126/science.aar7191 30115783

[ref15] Kumar A. Sharma S. Chunduri V. Kaur A. Kaur S. Malhotra N.. (2020). Genome-wide identification and characterization of heat shock protein family reveals role in development and stress conditions in *Triticum aestivum* L. Sci. Rep. 10:7858. doi: 10.1038/s41598-020-64746-2, PMID: 32398647 PMC7217896

[ref16] Li L. Mao X. Wang J. Chang X. Reynolds M. Jing R. (2019). Genetic dissection of drought and heat-responsive agronomic traits in wheat. Plant Cell Environ. 42, 2540–2553. doi: 10.1111/pce.13577, PMID: 31077401 PMC6851630

[ref17] Liu B. Asseng S. Liu L. Tang L. Cao W. Zhu Y. (2016). Testing the responses of four wheat crop models to heat stress at anthesis and grain filling. Glob. Chang Biol. 22, 1890–1903. doi: 10.1111/gcb.13212, PMID: 26725507

[ref18] Lu L. Liu H. Wu Y. Yan G. (2022). Wheat genotypes tolerant to heat at seedling stage tend to be also tolerant at adult stage: the possibility of early selection for heat tolerance breeding. Crop J. 10, 1006–1013. doi: 10.1016/j.cj.2022.01.005

[ref19] Mason R. E. Mondal S. Beecher F. W. Pacheco A. Jampala B. Ibrahim A. M. H.. (2010). QTL associated with heat susceptibility index in wheat (*Triticum aestivum* L.) under short-term reproductive stage heat stress. Euphytica 174, 423–436. doi: 10.1007/S10681-010-0151-X

[ref21] Maulana F. Ayalew H. Anderson J. D. Kumssa T. T. Huang W. Ma X. F. (2018). Genome-wide association mapping of seedling heat tolerance in winter wheat. Front. Plant Sci. 9:1272. doi: 10.3389/fpls.2018.01272, PMID: 30233617 PMC6131858

[ref22] Mishra S. Jha A. B. Dubey R. S. (2011). Arsenite treatment induces oxidative stress, upregulates antioxidant system, and causes phytochelatin synthesis in rice seedlings. Protoplasma 248, 565–577. doi: 10.1007/S00709-010-0210-0, PMID: 20857150

[ref23] Mittler R. Vanderauwera S. Suzuki N. Miller G. Tognetti V. B. Vandepoele K.. (2011). ROS signaling: the new wave? Trends Plant Sci. 16, 300–309. doi: 10.1016/j.tplants.2011.03.007, PMID: 21482172

[ref24] Mullarkey M. Jones P. (2000). Isolation and analysis of thermotolerant mutants of wheat. J. Exp. Bot. 51, 139–146. doi: 10.1093/jexbot/51.342.139, PMID: 10938805

[ref26] Rahaie M. Xue G. P. Schenk P. M. (2013). “The role of transcription factors in wheat under different abiotic stresses,” in *Abiotic Stress. Vol. 201*. eds. K. Vahdati and C. Leslie (IntechOpen), 367–385.

[ref27] Ram Poudel M. Bahadur Poudel P. Raj Puri R. Kumari Paudel H. (2021). Variability, correlation and path coefficient analysis for agro-morphological traits in wheat genotypes (*Triticum aestivum* L.) under Normal and heat stress. Nepjol. Info 9, 65–74. doi: 10.3126/ijasbt.v9i1.35985

[ref28] Sangwan S. Munjal R. Ram K. Kumar N. (2019). QTL mapping for morphological and physiological traits in RILs of spring wheat population of WH1021 9 WH711. JEB 40, 674–682. doi: 10.22438/jeb/40/4/MRN-1002

[ref30] Shahinnia F. Roy J. Le Laborde B. Sznajder B. Kalambettu P. Mahjourimajd S. (2016). Genetic association of stomatal traits and yield in wheat grown in low rainfall environments. BMC Plant Biol., 16, 150. doi: 10.1186/S12870-016-0838-9, PMID: 27378125 PMC4932692

[ref31] Tahmasebi G. Heydarnezhadian J. Aboughadareh A. P. (2013). Evaluation of yield and yield components in some of promising wheat lines. Int. J. Agric. Crop Sci. 5:2379.

[ref32] Talukder A. McDonald G. Gill G. S. (2014). Effect of short-term heat stress prior to flowering and early grain set on the grain yield of wheat. Field Crops Res. 160, 54–63. doi: 10.1016/j.fcr.2014.01.013

[ref33] Thomelin P. Bonneau J. Brien C. Suchecki R. Baumann U. Kalambettu P.. (2021). The wheat seven in absentia gene is associated with increases in biomass and yield in hot climates. J. Exp. Bot. 72, 3774–3791. doi: 10.1093/jxb/erab044, PMID: 33543261 PMC8096608

[ref34] Vijayalakshmi K. Fritz A. K. Paulsen G. M. Bai G. Pandravada S. Gill B. S. (2010). Modeling and mapping QTL for senescence-related traits in winter wheat under high temperature. Mol. Breed. 26, 163–175. doi: 10.1007/S11032-009-9366-8

[ref36] Zhai H. Jiang C. Zhao Y. Yang S. Li Y. Yan K.. (2021). Wheat heat tolerance is impaired by heightened deletions in the distal end of 4AL chromosomal arm. Plant Biotechnol. J. 19, 1038–1051. doi: 10.1111/pbi.13529, PMID: 33372381 PMC8131055

